# The influence of the 4th industrial revolution on family cohesion–A systematic review protocol

**DOI:** 10.1371/journal.pone.0288954

**Published:** 2023-09-08

**Authors:** Tolulope Victoria Balogun, Kezia Ruth October, Babatope Adebiyi, Nicolette V. Roman

**Affiliations:** The Centre for Interdisciplinary Studies of Children, Family and Society, Faculty of Community and Health Sciences, University of the Western Cape, Cape Town, South Africa; Manaaki Whenua - Landcare Research, NEW ZEALAND

## Abstract

Humanity may be facing untold threats and possible benefits as a result of the burgeoning 4th Industrial Revolution. New technologies introduced by the 4th Industrial Revolution have been purported to be gradually diminishing humans’ capacities like compassion and cooperation. Challenges such as security, trust, liability and personal data privacy issues are also being triggered which calls for stricter regulations. Emerging digital innovations allegedly attempts to widen the social and economic gap between the elites and the non-elites or the rich and the poor. Furthermore, other literature has pinpointed some of these propositions as skewed and biased, tending to ignore some other salient issues. The family, as a microcosm of a larger society, is certainly influenced by these technological interplay. It is therefore of great importance that keen attention is given to the family unit and its proper coherence and functioning within the sphere of the burgeoning terrain of the 4th Industrial Revolution. As such, this study seeks to undertake a systematic review by identifying, summarising and synthesising currently available research on the relationships between the emerging 4th Industrial Revolution and family cohesion.

## Introduction

Families are experiencing such peculiar challenges that have ever been recorded in the pages of history. The family, as a microcosm of a larger society, informs and influences the society or community and is also a pointer to societal challenges. The family is further seen as a social system that operates through social bonds. The family plays a pivotal role in cohesion through the social interactions within the society that influences social functioning and promotes economic and social development [1]. Family cohesion in this sense is characterised by social networks that are shared between family members and acts as a protective resource during challenging circumstances. Similar to family cohesion, social cohesion is a process that is present when shared social relations and interactions are present within a group, community or society [2]. However, a variety of social challenges impact the process and development of family cohesion and social cohesion. For example, poverty has been seen as a major cause of crime-ridden neighbourhoods, especially in South African communities and this has been identified as indicative of the lack of social cohesiveness [3].

[4] in a 2018 survey randomly administered to a sample of caregivers with 483 children under the age of 10, found that social cohesion mediated between the neighbourhood poverty level and the abuse rate of children. Suggestive evidence depicts that social cohesion is negatively correlated with service delivery protests, unemployment and poverty whilst social cohesion is positively correlated with employment, competence, social and economic development [5]. Furthermore, these findings may induce a significant consequence on the family environment, family functioning and family cohesion. It is therefore essential to examine the influencers of family cohesion and also to give proper attention to the proper functioning of the family unit in societies and communities, as they are drivers of social cohesion.

Within the technological, social and economic realm, humanity may be facing untold threats and possible benefits as a result of the burgeoning 4th Industrial Revolution (4IR). [6] suggests that new technologies introduced by the 4IR have gradually diminished humans’ capacities like compassion and cooperation. Challenges such as security, trust, liability and privacy issues with personal data, are also being triggered which calls for stricter regulation [7]. These adjustments may affect the broader social systems such as the functioning of family and social environments as emerging digital innovations are attempting to widen the social and economic gap between the elites and the non-elites or the rich and the poor, which may ultimately influence the sense of cohesion within the family and society.

A variety of factors may be influenced by the 4th Industrial Revolution (4IR). For example, the global economy is moving towards the adoption of AI solutions which will facilitate keen competition among workers or employees who possess the required critical or scarce skills to implement, manage and work with the newly introduced technologies. Taking the ecological approach towards the family and social environment, these emerging adoptions affect the functioning of the family and social environment, as changes in one system may affect changes in another system. It can be argued that families cannot function independently from the broader socio-economic sphere, which brings in another level, the economy, which can impact the social environment of the individual, the family as well as the overall community. For example, communities with high levels of social cohesion have been shown to have protective enablers for adolescent non-risk behaviour. “Communities with greater support networks were able to work collectively to implement social controls to protect community members from negative social influences” [8]. Extant studies in countries like Singapore have indicated a direct link between citizen participation and high levels of e-skills of citizens [9].

Furthermore, [10] argues that extant literature has viewed the 4IR with a skewed lens, as such, the proposed expected outcomes of the 4IR and other futuristic technologies are being tackled with inaction and indifference. The author foresights that it is the apprehensive, careless, and passive disposition that is held towards the 4IR and its outcomes that will most probably produce undesirable results.

Most discussions about the influence of the 4th Industrial Revolution on the society focus on employment and jobs. However, it is not limited to these alone. The 4th Industrial Revolution (4IR) will and is already impacting the way we live. These shifts and changes may affect the functioning of the family and social environment through newfound processes of knowledge, social and daily life adjustments. Furthermore, these changes may result in a weakened social cohesion among family members as well as within their neighbourhoods [11].

It is with these cardinal sentiments that this study aims to explore previous research findings on the relationships that are emerging between family cohesion and the emerging 4th Industrial Revolution.

### Research question

Consequently, the research question that answer is sought for is—What is the influence of the emerging 4th Industrial Revolution on Family Cohesion?

## Materials and methods

This systematic review protocol has been registered with the International Prospective Register of Systematic Reviews (PROSPERO). The registration number is CRD42020212793. Ethics approval has also been obtained from the Human Social Sciences Research Ethics Committee of the University of the Western Cape. The researchers will ensure trustworthiness through the credibility of data searches and inclusion.

To report on the findings of this study, the Preferred Reporting Items for Systematic Reviews and Meta-Analyses Protocols (PRISMA-P) checklist will be utilised [12]. This study is a systematic literature review that will utilize search engines, databases and journals as the study population.

### Inclusion and exclusion criteria

#### Participants

All included studies will consist of participants (family members) from all family types regardless of geographical location, country, race, culture and age that align with the key terms of the review.

#### Concept

Information older than 10 years is to be excluded from the review and only current literature conducted within a ten-year period will be reviewed [13]. Most importantly, a preliminary literature search indicated a sparsity of the 4IR literature before 2011. Literature indicates three previous revolutions: the First Industrial Revolution (1IR), the Second Industrial Revolution (2IR), and the Third Industrial Revolution (3IR). There was no reference made to the 4IR before 2011. This review will therefore utilize the 10-year time frame to cover every reference previously made to the 4IR.

As such, only literature conducted within the period of January 2012—December 2022 will be reviewed.

#### Language & context

All settings are considered relative to families and their environment, and therefore not limited to any specific country, religion, culture or gender. The review will include all English existing research articles, mixed-methods studies, qualitative literature and quantitative literature that reported on the influence 4th Industrial Revolution (4IR). Studies will be included if they are: 1) original research; 2) results of primary data; 3) in the English language; 4) published in peer-reviewed journals; and (5) examined the relationship of the 4IR on family cohesion or family functioning. [14] observed that the inclusion of only studies written in the English language is a commonly used concept for systematic reviews given the difficulties that could be encountered in translating and replicating the review.

#### Search strategy

The following databases that will be utilised in the systematic review are: African Journals Online, CINAHL, Emerlad e-journal Premier, masterfile Premier, Ebscohost Web (Academic search complete, ERIC, Medline, PsycARTICLES and SocINDEX), Sage journals Online, Science Direct, Sabinet, JSTOR, Springer Link and PubMed.

#### Search terms

The search terms will include: (1) family, (2) family functioning, (3) family cohesion, (4) family wellbeing, (5) cohesion, (6) fourth industrial revolution, (7) 4th industrial revolution, (8) 4IR, (9) industry 4.0, (10) artificial intelligence (AI), (11) robotics, (12) the Internet of Things (IoT), (13) 3D printing, (14) genetic engineering, (15) quantum computing, (16) cloud computing, (17) cyber-physical systems, (18) gene editing, (19) other technologies, (20) internet, (21) social media. These search terms will be combined in various forms for the selected databases using Boolean operators.

### Screening of studies

The screening of studies will be carried out based on the inclusion and exclusion criteria highlighted in this study. First, all the search results will be imported into a spreadsheet in which the duplicates will be deleted. Then, all the titles and abstracts will be checked for relevance to the eligibility criteria, and all non-relevant studies will be deleted. Furthermore, the body of the studies will also be read for relevancy. Two of the authors/reviewers will independently undertake the screening of all the included studies and the differences in opinion will be resolved with the third reviewer.

### Data extraction

The first author will extract data from the identified databases and articles based on the inclusion criteria. The second author will check if the extracted data are accurate. As such, the titles and abstracts of the retrieved studies will be independently screened by the two authors whilst any disagreement will be resolved with the third author.

The following information will be extracted from the identified studies: country of study, setting of the study, sample size, demographics of the participants, family type, study design, instruments used for data collection and details of the intervention. The standardized, adapted version of the data extraction tool by [15] will be used to extract the data from the included studies.

### Data synthesis

The authors will extract data from the identified articles. The authors will summarize the findings and characteristics of the included studies in a tabularised format. Subsequently, a narrative synthesis will be provided which will present the relationships and overall assessment of the variables being studied. Review findings will afterwards be disseminated through publications in peer-reviewed journals and conferences and seminars.

### Critical appraisal of identified studies

The methodological quality assessment tool was adapted from [16, 17]. The authors believe that this tool will be appropriate for this current review and it was adapted accordingly. The methodological quality appraisal tool was used to evaluate the sampling methods; reliability and validity of the measuring tool; relationship between the intervention and the considered variable; data source; response rate; adequate sample size; representative sampling method; and detailed description of participants and setting.

A possible inclusion within the review is considered when the methodological quality appraisal score is obtained as satisfactory or good.

### Outcomes

The outcome of the review will be to understand the relationship and influence of the 4IR on cohesion amongst family members. All original studies indicating the outcome of interest will be included whether the outcome was reported as a primary outcome or not.

## Preliminary literature review

### Family functioning

The word ‘Family’ is a frequently used word that has been traditionally referred to as two married people of both sexes and their children. Furthermore, it has been described as a “social group characterised by common residence, economic cooperation and reproduction. It includes adults of both sexes at least two of whom maintain a socially approved sexual relationship and one or more children” [18]. However, this generation with its peculiarities has expanded the definition of the family to include: “Societal groups of members related by blood (kinship), adoption, foster care or ties of marriage (extended families), including civil, customary, or religious marriages, or communal union, and it extends beyond any particular shared physical residence” [19].

The Department of Social Development furthermore identified three crucial priorities that should direct the core functioning of the family unit. These are: (1) promotion of healthy family life; (2) family strengthening; and (3) family preservation [19].

Families have been identified as an important, indispensable system that provides the child with the needed developmental nourishment, support, nurturing and socialization. The home environment and the environment are described as being crucial to a child’s functioning and well-being [20]. The World Health Organisation defines health and well-being as the following, “Health is a state of complete physical, mental and social well-being and not merely the absence of disease or infirmity” [21]. Children are dependent on the environment provided for their safety, care, health, education, growth and development, activities, nutritional needs, encouragement and support. For children, the behaviour, knowledge, skills and attitudes of parents or primary caregivers largely influence their health and well-being [22].

### Family cohesion

Family Cohesion is therefore seen as a functioning process that is characterised by family members, especially those who are living in supportive and organised families as they are much more likely to have increased self-confidence, social competence, more independence, with decreased scepticism and anxiety [1, 23]. It is seen as the affective, physical and emotional connection and attachment that family members have towards one another [24]. Furthermore, a study that examined the relationships between maternal psychological control, family environment and the psychological well-being of pre-adolescents, [25] indicated that the atmosphere sustained in the family could have either positive or negative implications for the psychological well-being of children. These findings suggest that family environment and psychologically controlling parenting predicts the psychological well-being of pre-adolescents. Perhaps psychologically controlling parenting could create an environment that would have more conflict and less cohesion between family members and thus influence the psychological well-being of children in the family. Similarly, [26] in a systematic review examining associations between health behaviours and parenting approaches, indicated that the engagement in health behaviours, which promotes overall health and well-being, was associated with autonomy, supportive parenting, parental encouragement, behaviour control, parental nurturance, warmth and responsiveness, and parental involvement. Essentially family research further stipulates that cognitive and socio-emotional skills developed during child and adolescent rearing are essential predictors of life course outcomes such as health, educational attainment and labour market performance [27]. This suggests that families displaying higher levels of social cohesion may further contribute to the successful functioning of society [19]. Social cohesion is viewed as a fundamental aspect of both the family and social environment.

### The 4^th^ industrial revolution (4IR)

The 4^th^ Industrial Revolution depicts a pivotal change in the way we relate with ourselves, work, live and run our lives in general. It is a turn of a new era in which amazing technological innovations will influence and greatly impact human development. These advancements merge the biological, physical, and digital worlds in different ways that produce amazing promises and possibly potential perils. The 4^th^ industrial revolution is characterized by information and communications technology, digitization, artificial intelligence, robotics and machine learning. Some decision-making may shift from humans to machines. This shift will undeniably produce societal changes that will have an intense impact on families and the society.

The term, 4^th^ Industrial Revolution or Industry 4.0 first originated in April 2011 as “Industrie 4.0” during the Hannover Trade Fair held in Germany. It was however formally announced in 2013. Industry 4.0 was introduced by the German government as a strategic high-tech initiative geared towards promoting, revolutionalising and advancing the computerization of the manufacturing industry [28].

Furthermore, in 2016, the World Economic Forum (WEF) executive chairman, Prof. Klaus Schwab [29]. The WEF chairman indicated that the first industrial revolution was characterised with steam power to mechanise production. The second utilized electric power to create mass production whilst the third industrial revolution used electronics and information technology to automate production. The 4^th^ industrial revolution is a build-up of the third industrial revolution [30].

[31] indicates that South Africa is one of the countries that has currently embraced the promotion of the 4IR as one of the strategies for promoting inclusive growth. Despite the new innovations and the advanced way of living that the 4IR is introducing, [29] has suggested that the 4th industrial revolution will likely introduce some other disruptive changes to the labour market. A new breed of technology-savvy employees and managers will possibly emerge as a result of the digital transformation and innovations of the 4th industrial revolution. Governments and organisations are now focused on developing future competencies and skills. Consequently, new jobs and tasks will emerge whilst some other professions will become obsolete or automated.

Schwab indicated that the 4IR will greatly impact our identity, our sense of privacy, consumption patterns, work and leisure, how we cultivate our skills and careers, meeting and nurturing relationships and definitely family lifestyle among other areas [32]. He furthermore emphasised that one crucial challenge that is being posed by these new information technologies is the lack of privacy. The constant connection to all the automated advancements may hinder and deprive us of the time to pause, reflect, and engage in meaningful and nurturing conversations and relationships. It is in the context of this, that this study seeks to understand the influence of the 4IR and the technological transformations on family cohesion.

## Supporting information

S1 Checklist PRISMA-P (Preferred Reporting Items for Systematic review and Meta-Analysis Protocols) 2015 checklist: Recommended items to address in a systematic review protocol*. (DOC)
